# Molecular Phylogenetic Variability of *Fasciola gigantica* in Iran

**Published:** 2019-04

**Authors:** Saber RAEGHI, Soheila ROUHANI, Majid FASIHI HARANDI, Adel SPOTIN, Sahar GHODSIAN

**Affiliations:** 1. Department of Laboratory Sciences, Maragheh University of Medical Sciences, Maragheh, Iran; 2. Department of Parasitology and Mycology, School of Medicine, Shahid Beheshti University of Medical Sciences, Tehran, Iran; 3. Research Center for Hydatid Disease in Iran, School of Medicine, Kerman University of Medical Sciences, Kerman, Iran; 4. Department of Parasitology, Faculty of Medicine, Tabriz University of Medical Sciences, Tabriz, Iran

**Keywords:** *F. gigantica*, Spermatogenesis, Genetic diversity, Mitochondrial genes

## Abstract

**Background:**

Fascioliasis is one of important zoonotic disease caused by *Fasciola gigantica* and *F. hepatica*. The final hosts of this parasite are ruminants and humans. Iran is one of the endemic areas in the world, about six million people at risk of infection. The aim of this study was to identify and determine the genetic diversity of *Fasciola* species in cattle after distinguish of their species.

**Methods:**

One hundred and seventeen liver specimens collected from naturally infected cattle in 5 geographical regions in 2014–2017. Flukes stained with Hematoxylin-Carmine dye to examine for the existence of sperm within seminal vesicles. DNA was extracted from each individual, and ITS1, ND1and CO1 genes were amplified using specific primers. For discrimination of *Fasciola* species, ITS1 PCR-RFLP was used based on digestion pattern of *RsaI* enzyme. Genetic analyses and diversity and neutrality indices estimated by Dnasp5 based on NDI.

**Results:**

Six nonspermic and 111 spermic flukes were diagnosed. All of nonspermic specimens were *F. gigantica* and collected from South East, South West and North West of Iran. Genetic haplotype diversity has been observed in *F. gigantica* based on ND1. *F_st_* value analysis showed that minimum and maximum genetic difference between Iranian *F. gigantica* with Bangladesh (*F*_st_ = 0.01414) and Egypt (*F*_st_ = 0.36653) respectively.

**Conclusion:**

It is the first report of existing of nonspermic *Fasciola*. High haplotype and nucleotide diversity could be due to ecological factors in life cycle, animal migration and coexisting of the final host of this parasite. Haplotype and nucleotide diversity of spermic *F. gigantica* in Iran and other countries in the world led to creating a variety of haplogroups.

## Introduction

*Fasciola gigantica* and *F. hepatica* are among the most crucial zoonotic parasites that found in all 5 continents especially where cattle and sheep are reared. Animals or people usually become infected by eating raw watercress or other water plants contaminated with immature parasite larvae named metacercariae (1). *F. gigantica* is reported mainly from Asia and Africa and causes economic losses of US$3 billion annually due to its impact on livestock production, thereby affecting the food industry worldwide (2, 3). In Iran, human fascioliasis has been accounted for from various areas and two episodes of human fascioliasis in 1987 and 1997, influencing more than a few a huge number of individuals in Gilan Province, Bandar Anzali region (4, 5). Animal fascioliasis is quite frequent and occurs generally in most areas of the state and their prevalence reaches around 50% in certain provinces (6). Moreover, climate change affected on number of Fascioliasis in recent years (7).

Body size and shape are one the conventional, customary and essential strategy to recognize of *Fasciola* species as morphological criteria, however, these prerequisites are not, for the most part, confided in light of the variable scope of the species (8). *Fasciola* is meiotically functional diploid and can produce sperm and temporary store produced sperm in the seminal vesicles that named as nonpermic fluke. Male reproductive organ is the common predominant characteristic of both species that intermediate *Fasciola* flukes, which have morphological characteristic intermediate between *F. hepatica* and *F. gigantica* with no sperm (nonspermic fluke) in their seminal vesicles and maybe some *F. gigantica*, have been found in Asian countries as nonspermic (9, 10).

However, DNA sequences of nuclear ribosomal transcribed spacers (ITS) and RFLP methods and sequences analyses of CO1 and ND1 as mitochondrial genes appear the intraspecific phylogenetic relations of *Fasciola* spp. (11–13).

Population genetic analyses are a way to find the origin, evolution in populations and helpful to mitigate against their spread (14). The husbandry and management of different farms have the potential to affect the population structure of parasites by influencing the movement of the definitive host and, therefore, *F. gigantica* (15).

There are some reports from Iran ruminant fascioliasis particularly in cattle and buffaloes based on geography, climate variability and characterization of them according to molecular and phylogenetic methods (4, 16, 17). Likewise, there have been no rich and important overviews on molecular and spermatogenetic ability of *Fasciola*.

This study aimed to distinguish of *F. gigantica* spermatogenesis ability and, and in addition investigate their phylogenetic and diversity network and association with haplotypes from different parts of the world using mitochondrial (ND1) marker.

## Materials and Methods

### Study Population

**Fig. 1:**
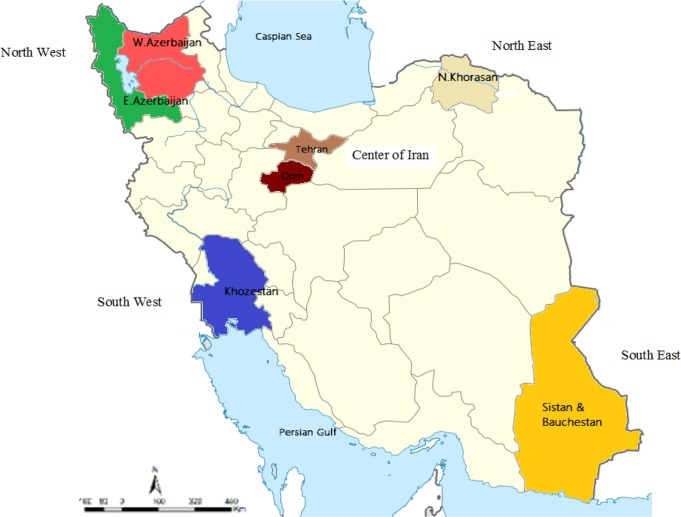
Sampling area of *F. gigantica* in this study from Iran

This cross-sectional study was performed on *Fasciola* spp. isolated from naturally hosts (cattle) in five regions from abattoirs in Iran from Jan 2015 to Dec 2017. Iran has different climates in different regions. The climate is influenced by Iran’s location between the subtropical aridity of the Arabian Desert areas and the subtropical humidity of the eastern Mediterranean area. About 70% percent of the average rainfall in the country falls between November and March (Fig. 1).

### Sampling of Fasciola and morphological analysis for spermatogenesis status

One hundred and seventeen liver samples from cow that were naturally infected were collected from different slaughterhouses in Iran.

This study was ethically approved by Shahid Beheshti University of Medical Sciences (SBMU).

One fluke of them isolated and the washed in 0.9% saline solution and fixed in 70% ethanol between two glass slides, and then measurement morphological criteria such as body length and width were carried out. The whole body of *Fasciola* including seminal vesicle in the anterior part of the worm was stained with haematoxylin carmine solution and observed under an optical microscope to examine for the existence of sperm (18). Prior to staining, a small posterior part of the fluke was used for DNA extraction.

### DNA extraction and amplification

Total DNA was extracted from individual *Fasciola* sample using High Pure PCR Template Preparation Kit (Dynabio®, Takapouzist, Iran), according to the manufacturer’s instructions and stored at −20 °C until use. ITS1 region as a nuclear marker was amplified with primers named ITS1-F and ITS1-R and fragments of each mitochondrial target region (ND1 & and CO1) were amplified by polymerase chain reaction (PCR) using designed primers (19). Total volume of the reaction was 40μl containing 4μl DNA template, 14 μl distilled water, 10 pmol of each primer, and 10 μl master mix (amplicon®). Reaction cycles consisted of an initial denaturing step at 94 °C for 90 sec, followed by 35 cycles at 94 °C for 90 sec, 53 °C (ITS1) or 55 °C (ND1 & CO1) for 90 sec and 72 °C for 120 sec, with a final extension at 72 °C for 10 min using a gradient thermocycler. DNA fragments were analyzed by 1.5% agarose gel electrophoresis

### PCR-RFLP method

ITS1 marker was used to identify different genus of *Fasciola* in this study. Briefly, the reaction level of 10 μL contained 5 μL of PCR products with approximately 680-bp fragments, 1 U of the RsaI restriction enzyme, and 1 μL of manufacturer-supplied reaction buffer (Cinagen®, Iran). After incubation at 37 °C for 3 h and heat inactivation of *Rsa*I at 65 °C for 15 min, the digested DNA samples were analyzed by gel electrophoresis (20).

### Sequences and phylogenetic analysis and genetic diversity indices

Products of ITS1, ND1 & CO1 of isolates sequenced by Bioneer Company using the same primers, used in the PCR. The sequences were aligned and compared with those of existing sequences from the region, related to *Fasciola* spp. available data from Iran and other countries deposited in GenBank using the bioinformatics multiple alignments. All characters equally weighted and alignment gaps were treated as missing data. Mitochondrial sequences (ND1) haplotype networks designed by popART-1.7 software. Diversity indices (Haplotype diversity; Hd and Nucleotide diversity: π) and neutrality indices (Tajima’s D and Fu’s Fs tests) were estimated by DnaSP software package version 5.10 (21). The degree of gene flow (gene migration) among the populations was evaluated using a pairwise fixation index (*F_st_*) (22).

## Results

### Microscopic observation

Both spermic and nonspermic *Fasciola* were detected in Iran. Six nonspermic flukes from South East, South west and Northwest of Iran obtained. Remaining *Fasciola* was spermic and detected from all five regions. Length to width ratio in the nonspermic *Fasciola* was as a morphological criterion of *F. gigantica*.

### Molecular findings

The amplicons of ITS1 (approximately 680 bp) that obtained from all of the spermic and nonspermic flukes cut using RsaI endonuclease digestion. RFLP pattern for nonspermic and spermic *F. gigantica* are 360,170 and 60. ND1 fragments (approximately 535 bp) and CO1 fragments (approximately 438 bp) were amplified for all specimens. We lost DNA of one nonspermic *Fasciola* because of staining haematoxylin before extracting DNA.

### Network and genetic diversity

**Fig. 2:**
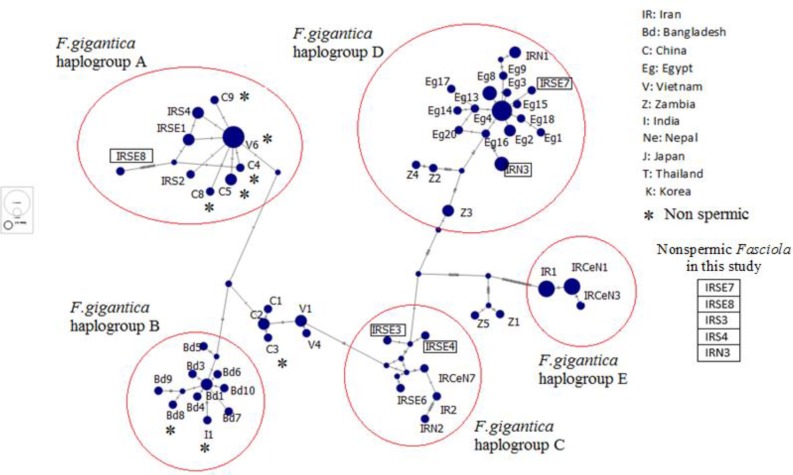
Mitochondrial sequences (ND1) haplotype networks in spermic and nonsermic haplotypes of *F. gigantica* from 5 different geographical regions of Iran using papART software. Nonspermic *Fasciola* show in rectangle and diameter with respect to the number of samples

Haplotype diversity, nucleotide diversity and Neutrality indices of *F. gigantica* flukes in this study compared with other countries based on ND1 gene shown in Table 1. The nucleotide sequences for each haplotype were deposited in GenBank. Mitochondrial sequences (ND1) haplotype networks in spermic and nonsermic haplotypes of *F. gigantica* from 5 different geographical regions of Iran showed in Fig. 2. Pairwise fixation index (*F_st_* values) between different *F. gigantica* populations in five geographical regions calculated by DnaSP software package with the nucleotide data set of ND1 gene (Table 2).

**Table 1:** Haplotype diversity and nucleotide diversity of nonspermic and spermic *Fasciola* fluke in Iran based on NDI gene

***Population***		***Diversity indices***				***Neutrality indices***	
		***N***	***Nh***	***Hd ± SD***	***π***	***Tajima’s D***	***Fu’s Fs statistic***
Nonspermic	This study	5	4	0.900 ± 0.025	0.03258	0.404	2.511
*F. gigantica*	(Iran)						
	Nepal	61	2	0.033 ± 0.031	0.00006	−1.082	−1.082
	China	121	5	0.081 ± 0.034	0.00015	−1.708*	−7.128**
	Myanmar	7	1	0.000 ± 0.000	0.00000	NC	NC
	East India	33	2	0.061 ± 0.056	0.00011	−1.008*	−5.338**
	Bangladesh	127	1	0.000 ± 0.000	0.00000	0.000	0.000
Spermic	This study	33	14	0.902 ± 0.030	0.04245	0.26421	5.563
*F. gigantica*	(Iran)						
	Nepal	20	10	0.758 ± 0.101	0.00366	−2.103*	−4.710**
	China	34	13	0.861 ± 0.039	0.00454	−0.945	−4.967**
	Myanmar	80	19	0.631 ± 0.061	0.00519	−1.379	−6.679**
	East India	91	32	0.751 ± 0.050	0.00242	NC	NC

N: number of flukes used to calculation, Nh: number of haplotypes, Hd: haplotype diversity, SD: standard deviation, π: nucleotide diversity, NC: Not Calculated

* Significant *P*-value (*P*<0.05)

** Significant *P*-value (*P*<0.02)

**Table 2:** Pairwise fixation index (*F*_st_ values) between different *F. gigantica* populations calculated from the nucleotide data set of NDI gene

***Population***	***Iran***	***Egypt***	***Zambia***	***Bangladesh***	***China***
Iran	-	-	-	-	-
Egypt	0.36653	-	-	-	-
Zambia	0.15576	0.42757	-	-	-
Bangladesh	0.01414	0.35009	0.15172	-	-
China	0.13280	0.67298	0.38275	0.11801	-
Vietnam	0.28069	0.03508	0.51872	0.15172	0.03508

## Discussion

Fascioliasis is one of the most imperative worry for both general wellbeing angles and veterinary issues. The separation of *Fasciola* species is essential on the grounds that their epidemiological patterns as far as both species of *Fasciola* existent in Iran in various hosts (23). The aim of this study was to identify and determine the genetic diversity of *Fasciola* species in cattle after distinguish of their species. All of one hundred and seventeen flukes recognized as *F. gigantica* by described morphometric items (8), but 6 of them observed as nonspermic by microscopy observation after staining, that obtained from South East of Iran, near the border of Pakistan and South West of Iran that traditionally nurtured.

The proportion of body length and width (BL/BW) is one of the valuable criteria for separation of species in *Fasciola* (24). A morphological report from north of Iran indicating that existence of intermediate forms of *Fasciola* (8), but our morphological results do not show the significant difference in morphometric items between spermic and nonspermic as intermediate flukes. Notwithstanding morphological features are not suitable or appropriate for identification because of coexistence of *Fasciola* species like in Iran (25, 26). However, we used molecular methods for discrimination of nonspermic *Fasciola* by ITS1-RFLP and affirmed them by CO1 sequences analysis as *F. gigantica.*

Nonspermic *Fasciola* has been reported already from many Asian countries and there is genetic similarity of these flukes with spermic *F. gigantica*. Although some hypothesis demonstrated that nonspermic flukes had probably retained or lost their spermatogenetic ability or capacity (9, 18).

*Fasciola* sp. taxa in Southwest of Asia in India and Bangladesh near of this region of Iran, however gigantica type of these flukes detected in nonspermic *Fasciola* sp. using ITS-RFLP, and also phylogenetic study in both ND1 and CO1 genes showed that they placed in *F. gigantica* complex (9, 10).

In this study, diversity indices of *F. gigantica* are high in both spermic and nonspermic flukes. Neutrality indices show that there is high polymorphism in *F. gigantica* haplotyps of Iran, and coalescence in population based on ND1 gene. Moreover, we could not determine these indices using CO1 gene because unavailability CO1 sequence data in GenBank from other countries.

Median-joining network algorithm of haplotypes based on ND1 gene obtained from *F. gigantica* from Iran and records in GenBank from Japan, Korea, China, Vietnam, Thailand, Nepal, India, Egypt and Zambia, show 5 haplogroups (Fig. 2). Nonspermic samples of this study distributed in haplogrop A (IRSE8), D (IRSE7, IRN3) and C (IRS3, IRS4). IRSE8 in haplogroup A is close to nonspermic haplotype from China, Vietnam, Nepal, Bangladesh, Korea and Japan. This sample nonspermic specimen is one of intermediated forms of *Fasciola* in spite of morphometric criteria. Because of low number flukes, we cannot judge about it now.

Also IRS3 & IRS4 in group C and IRSE7& IRN3 in group D, isolated from South East and North West of Iran, respectively located beside of another spermic haplotype from Iran.

These flukes likely considered as irregular *F. gigantica* with oligozoospermia happened in light of the maturing of flukes. This finding appeared in Mohanta from Bangladesh before (18).

In addition, the origin of C and D haplogroups are not clear, because it has never been detected in any of the references in other countries. Some difficulty in haplotype detection, because of haplotype novelty from Java in Indonesia was reported (9). Maybe this novelty is due to hosts of *Fasciola.* Iran is a vast country and multiple factors may affect genetic variation and haplotypes. Population genetic structures in of *F. gigantica* in Iran is near to Bangladesh (*F_st_*=0.01414) and the most distant population genetics with Egyptian (*F_st_*=0.36653) based on ND1 gene. This variation and big gene drift show distribution of *F. gigantica* population that caused by host and different climates in Iran. Our hypothesis is that origin of *F. gigantica* of Iran and Bangladesh is common that additional studies required.

Molecular phylogeny with mtDNA, including ND1 and CO1, can be effectively used for proper differentiation of haplotypes, (11, 23) but using other genetic marker and method like *pepck* gene and MLST help understand and decryption of genetic history of *Fasciola* (9).

## Conclusion

Nonspermic *F. gigantica* found in Iran. Different genetic structures through the other *Fasciola* population in the world according to genetic indices seen, but to complete and find genetic diversity and history, other molecular studies with large sample size from other regions of Iran with different climates is necessary.

## Ethical considerations

Ethical issues (Including plagiarism, informed consent, misconduct, data fabrication and/or falsification, double publication and/or submission, redundancy, etc.) have been completely observed by the authors.
